# A peptide blocking the ADORA1-neurabin interaction is anticonvulsant and inhibits epilepsy in an Alzheimer’s model

**DOI:** 10.1172/jci.insight.155002

**Published:** 2022-06-08

**Authors:** Shalini Saggu, Yunjia Chen, Liping Chen, Diana Pizarro, Sandipan Pati, Wen Jing Law, Lori McMahon, Kai Jiao, Qin Wang

**Affiliations:** 1 Departments of Cell, Developmental and Integrative Biology,; 2 Department of Neurology, and; 3 Department of Genetics, University of Alabama at Birmingham, Alabama, USA.

**Keywords:** Neuroscience, Therapeutics, Epilepsy, G protein&ndash;coupled receptors, Pharmacology

## Abstract

Epileptic seizures are common sequelae of stroke, acute brain injury, and chronic neurodegenerative diseases, including Alzheimer’s disease (AD), and cannot be effectively controlled in approximately 40% of patients, necessitating the development of novel therapeutic agents. Activation of the A1 receptor (A1R) by endogenous adenosine is an intrinsic mechanism to self-terminate seizures and protect neurons from excitotoxicity. However, targeting A1R for neurological disorders has been hindered by side effects associated with its broad expression outside the nervous system. Here we aim to target the neural-specific A1R/neurabin/regulator of G protein signaling 4 (A1R/neurabin/RGS4) complex that dictates A1R signaling strength and response outcome in the brain. We developed a peptide that blocks the A1R-neurabin interaction to enhance A1R activity. Intracerebroventricular or i.n. administration of this peptide shows marked protection against kainate-induced seizures and neuronal death. Furthermore, in an AD mouse model with spontaneous seizures, nasal delivery of this blocking peptide reduces epileptic spike frequency. Significantly, the anticonvulsant and neuroprotective effects of this peptide are achieved through enhanced A1R function in response to endogenous adenosine in the brain, thus, avoiding side effects associated with A1R activation in peripheral tissues and organs. Our study informs potentially new anti-seizure therapy applicable to epilepsy and other neurological illness with comorbid seizures.

## Graphical abstract

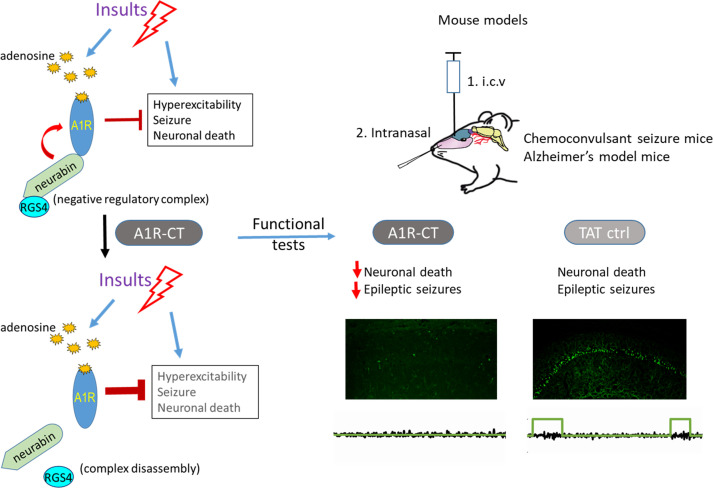

## Introduction

Epileptic seizures are common consequences of stroke and brain injury and often a comorbidity in chronic neurodegenerative diseases (1, 2). In Alzheimer’s disease (AD) in particular, the most common type of dementia affecting nearly fifty million people globally, the prevalence of seizure reaches up to 64% (3, 4). Left uncontrolled, seizures can cause substantial neural injury and cognitive impairment (5, 6). Despite progress in anti-seizure drug (ASD) development, approximately 40% of patients with epilepsy remain refractory to existing therapies (1, 7). Currently available anticonvulsants are often poorly tolerated and less effective in controlling seizures in AD patients (3, 8). Therefore, there is an urgent unmet need to discover novel therapeutic interventions for treatment of epileptic seizures.

Seizures and epileptic paroxysms induce a massive release of adenosine in the brain, which has been well established as the key endogenous defense mechanism to self-terminate seizures through activation of the most abundant A1 subtype of adenosine receptors (A1R) (9–13). Throughout the brain, activation of A1R limits neuronal excitability through coupling to K^+^ channels causing hyperpolarization and indirectly by decreasing glutamate release (14–16). Additionally, A1R has recently been identified as a critical player mediating the negative feedback control of neuronal activity by microglia (17). Significantly, activation of A1R effectively suppresses seizures that are resistant to most ASDs (18). In addition to seizure control, activation of A1R protects against neuronal damage from hypoxia and ischemia (19) and is responsible for deep brain stimulation-mediated attenuation of tremor (20). Despite its therapeutic effectiveness against various disease conditions, targeting the A1R for neurological disorders has been extremely challenging due to the broad expression of this receptor outside the nervous system in multiple tissues and organs including the heart, lung, and kidney. Thus, when using adenosine or A1R-directed ligands, peripheral actions evoked by these drugs will diminish the therapeutic gains achieved for central disease conditions. To date, there are no effective drugs that target A1R for seizure control and neuroprotection.

A1R belongs to the GPCR superfamily. We have previously identified an essential mechanism that controls the strength of A1R/G_i/o_ signaling responses and A1R-mediated anticonvulsant and neuroprotective effects (21, 22). A1R directly interacts with neurabin, a neural tissue-specific scaffolding protein. Neurabin scaffolds the complex formation between the active A1R and the regulator of G protein signaling 4 (RGS4) to diminish A1R-induced signaling responses through G_i/o_ proteins (21, 22). Based on our previous discoveries, we propose that disruption of the A1R-neurabin interaction would enhance A1R signaling and boost A1R-mediated anti-seizure and neuroprotective effects in response to endogenously released adenosine in the injured brain without activating A1R systemically, thus, avoiding peripheral side effects.

We now demonstrate that neurabin acts as a sensitive rate-limiting factor for modulation of A1R-elicited responses, supporting the feasibility of disruption of the A1R-neurabin interaction as a means to enhance endogenous adenosine-elicited anti-seizure response through A1R. Furthermore, we develop a peptide consisting of the A1R C-terminal sequence (referred to as the A1R-CT peptide) that disrupts A1R-neurabin interaction and reverses neurabin-mediated attenuation of A1R signaling. Excitingly, this blocking peptide exhibits strong anticonvulsant and neuroprotective effects against kainate-induced seizures when administered through intracerebroventricular or i.n. delivery. Finally, we show that i.n. administration of the A1R-CT peptide also effectively reduces epileptic activities in an AD mouse model. Our study informs new treatment for epileptic seizures under pathological conditions.

## Results

### The A1R-mediated anticonvulsant effect is regulated by the neurabin/RGS4 complex and is particularly sensitive to a change in neurabin levels.

**Figure 1:**
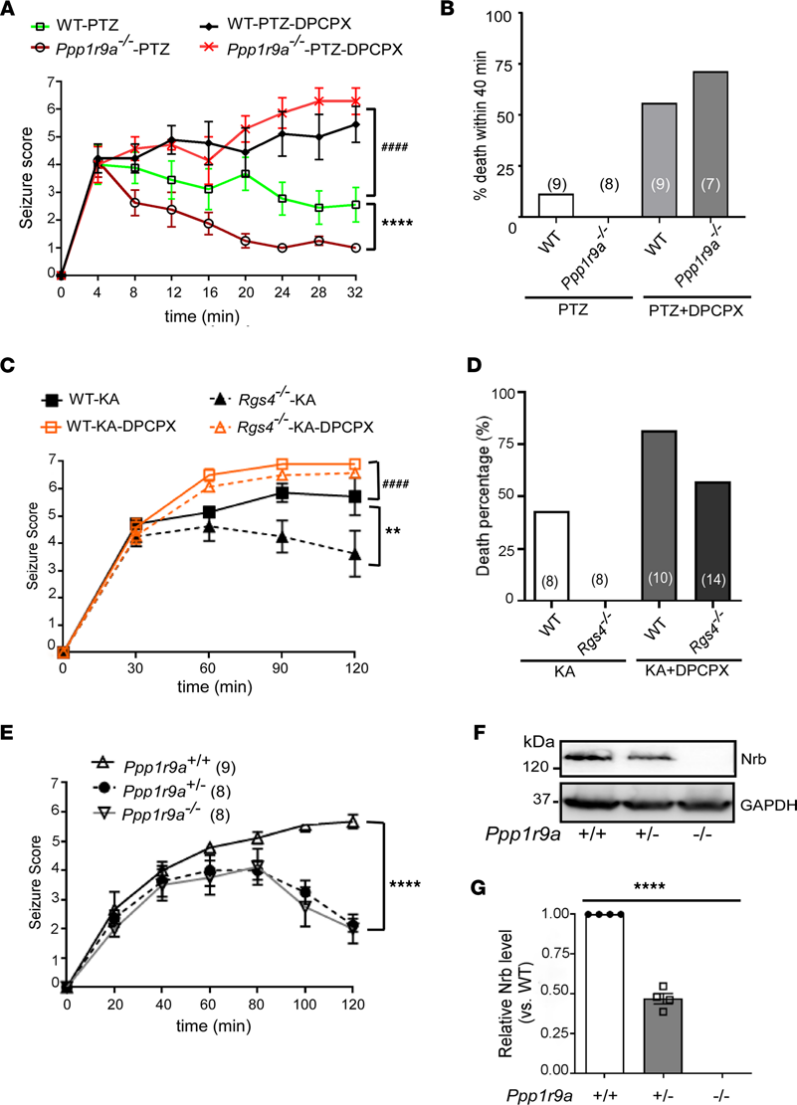
The A1R-mediated anticonvulsant effect is regulated by neurabin and RGS4 and is particularly sensitive to the change in neurabin levels. (**A**) Neurabin deficiency (*Ppp1r9a^–/–^*) attenuates seizure severity in response to PTZ. Seizure activity over time following PTZ injection was scored. *n* = 8–9/group. ****P < 0.0001, *Ppp1r9a^–/–^* versus WT mice by 2-way ANOVA. ^####^*P* < 0.0001, WT mice treated with PTZ alone versus PTZ plus DPCPX by 2-way ANOVA. (**B**) PTZ-induced lethality within 40 minutes following injection is recorded in mice examined in A. The number of mice in each group is indicated in parentheses. (**C**) Seizure severity in response to kainate is measured in the *Rgs4^–/–^* mouse line and its corresponding WT line. *n* = 8–14/group. **P < 0.01, *Rgs4^–/–^* versus WT mice. ^####^*P* < 0.0001, WT mice treated with kainate alone versus kainate plus DPCPX by 2-way ANOVA. (**D**) Kainate-induced lethality is recorded in mice examined in **C**. Data are expressed as percentage of death in WT and *Rgs4^–/–^* mice caused by administration of kainate alone or with DPCPX. The number of mice in each group is indicated in parentheses. (**E**) A1R-mediated anticonvulsant effects are enhanced in mice with reduced neurabin expression. Seizure severity in response to kainate is attenuated in both *Ppp1r9a^+/–^* and *Ppp1r9a^–/–^* mice. The number of mice in each group is indicated in parentheses. ****P < 0.0001, *Ppp1r9a^+/–^* versus *Ppp1r9a^+/+^* mice by 2-way ANOVA. (**F** and **G**) Expression of neurabin in brain lysates of *Ppp1r9a^+/+^*, *Ppp1r9a^+/–^*, and *Ppp1r9a^–/–^* mice. (**F**) Representative Western blots. (**G**) Quantitation of expression levels of neurabin in the brain of mice with indicated genotypes. ****P < 0.0001 by 1-way ANOVA. *n* = 4/group. Data are presented as mean ± SEM. See complete unedited blots in the supplemental material; supplemental material available online with this article; https://doi.org/10.1172/jci.insight.155002DS1.

We have previously identified an A1R/neurabin/RGS4 complex that downregulates A1R signaling in response to endogenous adenosine (21, 22). Consistently, the A1R-mediated anticonvulsant effect against kainate insult was enhanced in neurabin-deficient (*Ppp1r9a^–/–^*) mice (21). Here we tested this mechanism in another chemoconvulsant model, pentylenetetrazol-induced (PTZ-induced) seizure. PTZ is a GABA_A_ receptor antagonist that induces a rapid seizure response, which peaked at 4 minutes and lasted over 30 minutes in WT mice (Figure 1A). About 10% of PTZ-treated WT mice died within 40 minutes (Figure 1B). In *Ppp1r9a^–/–^* (neurabin-null) mice, PTZ-induced seizures quickly declined over time after the initial peak (Figure 1A), and all mice survived (Figure 1B). When mice were cotreated with an A1R-selective blocker, 8-Cyclopentyl-1,3-dipropylxanthine (DPCPX), PTZ-induced seizures in both WT and neurabin-null mice continued to progress to a much more severe level than those induced by PTZ alone. There was no significant difference between the 2 genotypes (Figure 1A), suggesting the requirement of A1R activation in reducing the seizure severity in neurabin-null mice. The combined treatment with PTZ and DPCPX led to more than 55% deaths in both genotypes (Figure 1B). These data clearly demonstrate the critical role of neurabin in attenuating the A1R-mediated anticonvulsant effect. In mice without neurabin expression, the A1R-dependent protective effect is significantly enhanced.

Given that neurabin scaffolds RGS4 recruitment to A1R to downregulate G protein signaling (21, 22), we predicted that the A1R-mediated anticonvulsant response would be enhanced in RGS4-null (*Rgs4^–/–^*) mice (23–25). Indeed, in *Rgs4^–/–^* mice, kainate-induced seizures were significantly attenuated in both extent and duration compared with WT mice (Figure 1C). While kainate administration at 20 mg/kg resulted in over 40% mortality in WT mice, all *Rgs4^–/–^* mice survived the same insult (Figure 1D). Furthermore, blockade of A1R by DPCPX increased the seizure severity and mortality rate in *Rgs4^–/–^* mice to a level similar to that in WT mice (Figure 1, C and D), suggesting the requirement of A1R activity in protecting *Rgs4^–/–^* mice against seizures.

Our previous mechanistic studies have suggested a central rate-limiting role of neurabin in regulating A1R signaling (22), which motivated us to test whether the A1R-mediated anticonvulsant effect is sensitive to the change in neurabin levels. We therefore examined kainate-induced seizure in neurabin-heterozygous (*Ppp1r9a^+/–^*) mice. Strikingly, the severity and progression of kainate-induced seizures in *Ppp1r9a^+/–^* mice were reduced to a level similar to that in *Ppp1r9a^–/–^* mice (Figure 1E). We confirmed that, in heterozygous mice, the neurabin expression level was about 50% of the WT level (Figure 1, F and G). In contrast, a 50% reduction in RGS4 levels has no effect on the A1R-dependent anticonvulsant response (average seizure severity scores are 5.7 and 5.6 in WT and *Rgs4^+/–^* mice, respectively; *n* = 5–8/group). These data suggest that the A1R-mediated anticonvulsant effect is particularly sensitive to neurabin-mediated regulation; a 50% reduction in neurabin levels can lead to a sufficient enhancement of the A1R-mediated anti-seizure effect. These data also strongly support that disruption of the A1R-neurabin interaction would be an effective means of seizure control.

### Neurabin and RGS4 attenuate A1R-mediated inhibition of synaptic transmission.

**Figure 2:**
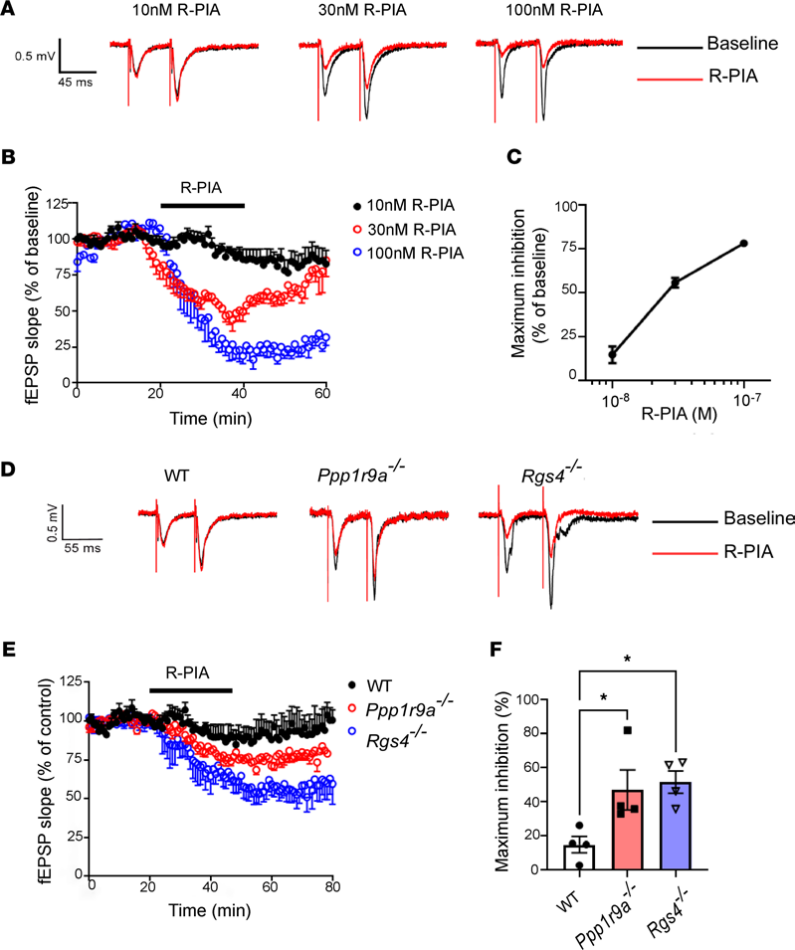
A1R-induced inhibition of synaptic transmission is enhanced in mice without neurabin or RGS4 expression. (**A**) Representative traces of fEPSP at CA3-CA1 synapses in hippocampal slices from WT mice treated with different concentrations of R-PIA. (**B**) Summary plot of mouse hippocampal slices exposed to R-PIA (min 20–50) showing the reduction of the fEPSP during bath application of 10 nM (*n* = 5), 30nM (*n* = 4), and 100nM (*n* = 4) R-PIA in WT brain slices. The amplitude of fEPSP slope was normalized to its baseline amplitude. (**C**) Statistical inhibitory effect of fEPSP slope by 10 nM, 30 nM, and 100 nM R-PIA on slices from WT mice. (**D**) Representative traces of fEPSP recorded during a 180 ms (at bar) in slices from WT, neurabin-deficient (*Ppp1r9a^–/–^*), and RGS4-deficient (*Rgs4^–/–^*) mice. (**E**) Summary plot of mouse hippocampal slices exposed to 10nM R-PIA (min 20–50) showing a larger magnitude depression in *Ppp1r9a^–/–^* and *Rgs4^–/–^* mice than in WT mice. The amplitude of fEPSP slope was normalized to its baseline amplitude. *n* = 4/group. (**F**) Summary of inhibition of 10 nM R-PIA on slices from WT, *Ppp1r9a^–/–^*, and *Rgs4^–/–^* mice, respectively. *P < 0.05 by 1-way ANOVA multiple comparisons. *n* = 4/group. Data were shown as mean ± SEM.

Adenosine A1R elicits anticonvulsant effects through the inhibition of synaptic transmission (16). As shown in Figure 2, A–C, an A1R agonist, (−)-N^6^-(2-Phenylisopropyl)adenosine (R-PIA), dose-dependently inhibited field excitatory postsynaptic potential (fEPSP) at the hippocampal CA3-CA1 synapses in WT mice. In total, 100 nM R-PIA resulted in about 75% inhibition of baseline transmission (Figure 2, B and C). We next sought to examine the impact of loss of neurabin or RGS4 expression on A1R-mediated depression of synaptic neurotransmission in hippocampal slices. In hippocampal slices prepared from *Ppp1r9a^–/–^* or *Rgs4^–/–^* mice, the same concentration of R-PIA induced a significantly higher level of inhibition of fEPSP at the CA3-CA1 synapse than in hippocampal slices from WT mice (Figure 2, D–F). These data demonstrate that A1R-mediated synaptic inhibition is enhanced in the absence of neurabin and RGS4 expression. Such enhancement would underlie the greater anticonvulsant effects by A1R in mice lacking these proteins.

### Neurabin reduces both the response sensitivity and duration of A1R-mediated neuroprotective Akt signaling in neurons.

**Figure 3:**
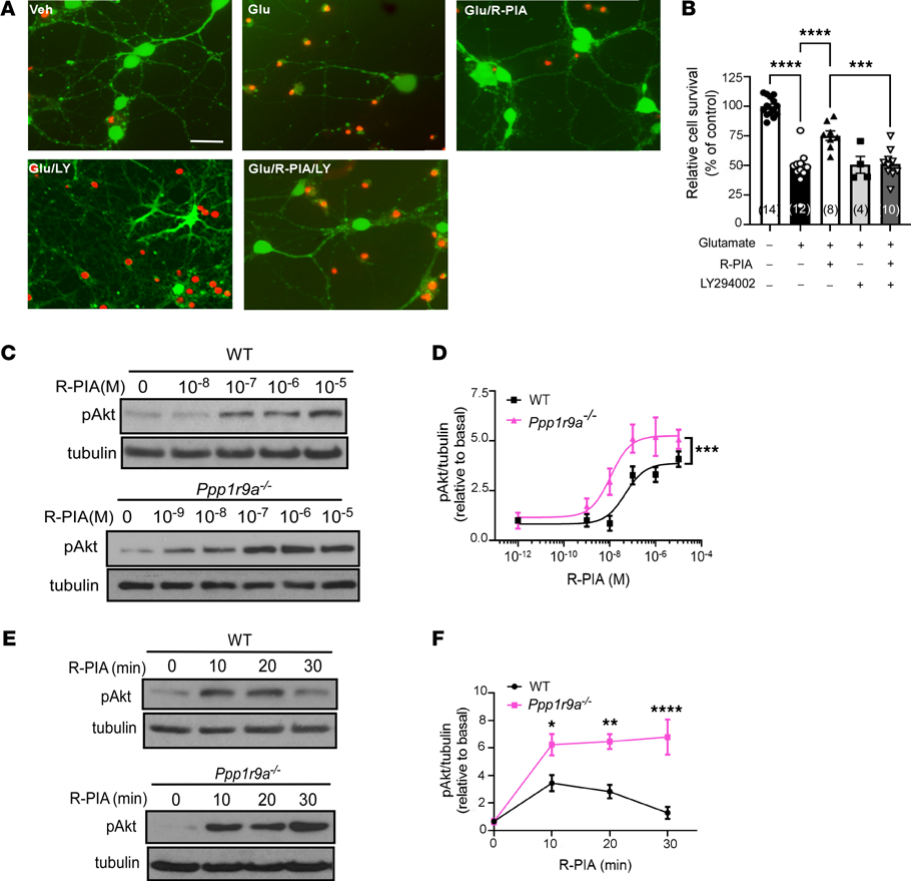
Neurabin deficiency enhances A1R-mediated neuroprotective Akt signaling in neurons. (**A**) *P*I3K/Akt signaling is required for A1R-mediated neuroprotection against glutamate excitotoxicity. WT hippocampal neurons (13–14 DIV) were treated under indicated conditions. Representative images of live (green) and dead (red) neurons are shown. (**B**) Quantitation of cell survival under indicated treatment. ***P < 0.001; ****P < 0.0001 by 1-way ANOVA multiple comparisons. Sample numbers are indicated in parentheses. (**C**) Neurabin deficiency enhances A1R-mediated Akt activation in neurons. Primary hippocampal neurons derived from WT or neurabin deficiency (*Ppp1r9a^–/–^*) mice were treated with R-PIA at the indicated concentrations. Representative Western blots are shown. (**D**) Quantitation of phospho-Akt (pAkt, Thr308). ***P < 0.001 by 2-way ANOVA. *n* = 4/group. (**E**) Neurabin deficiency prolongs the A1R-mediated Akt response in neurons. WT and *Ppp1r9a^–/–^* neurons were stimulated with 1 μM R-PIA for the indicated time durations. Representative blots are shown. (**F**) Quantitation of pAkt levels. *P < 0.05; **P < 0.01; ****P < 0.0001 by 2-way ANOVA multiple comparisons. *n* = 4/group. Data were shown as mean ± SEM. Scale bar: 20 μm.

The serine-threonine protein kinase Akt is an important neurotrophic signaling component that exerts protection against neuronal death (26). In primary cultured hippocampal neurons, an inhibitor of PI3K/Akt signaling, LY294002, abolished the protective effect of the A1R agonist R-PIA on glutamate-induced neuronal death (Figure 3, A and B), indicating the requirement of the PI3K/Akt pathway in A1R-elicited neuroprotection. We further examined A1R-mediated Akt activation in primary neurons derived from WT and neurabin-deficient (*Ppp1r9a^–/–^*) mice. In WT neurons, stimulation of A1R led to a dose-dependent increase of Akt phosphorylation at threonine 308 (Thr 308) in the activation loop. In neurons lacking neurabin expression, the same dose of R-PIA induced a significantly higher level of Akt phosphorylation compared with that in WT neurons (Figure 3, C and D), suggesting that the response sensitivity of A1R in inducing Akt signaling is enhanced in the absence of neurabin expression. Furthermore, A1R-mediated Akt activation was markedly prolonged in neurons without neurabin expression compared with WT neurons. While Akt activation was desensitized after 30 minutes of stimulation with R-PIA in WT neurons, no apparent reduction in phospho-Akt (i.e., active Akt) levels was observed at the 30-minute time point in neurons lacking neurabin (Figure 3, E and F). Taken together, these data demonstrate that neurabin reduces A1R-dependent neuroprotective Akt signaling in neurons.

### The A1R-CT peptide blocks the A1R-neurabin interaction and abolishes neurabin-mediated inhibition of A1R signaling.

**Figure 4:**
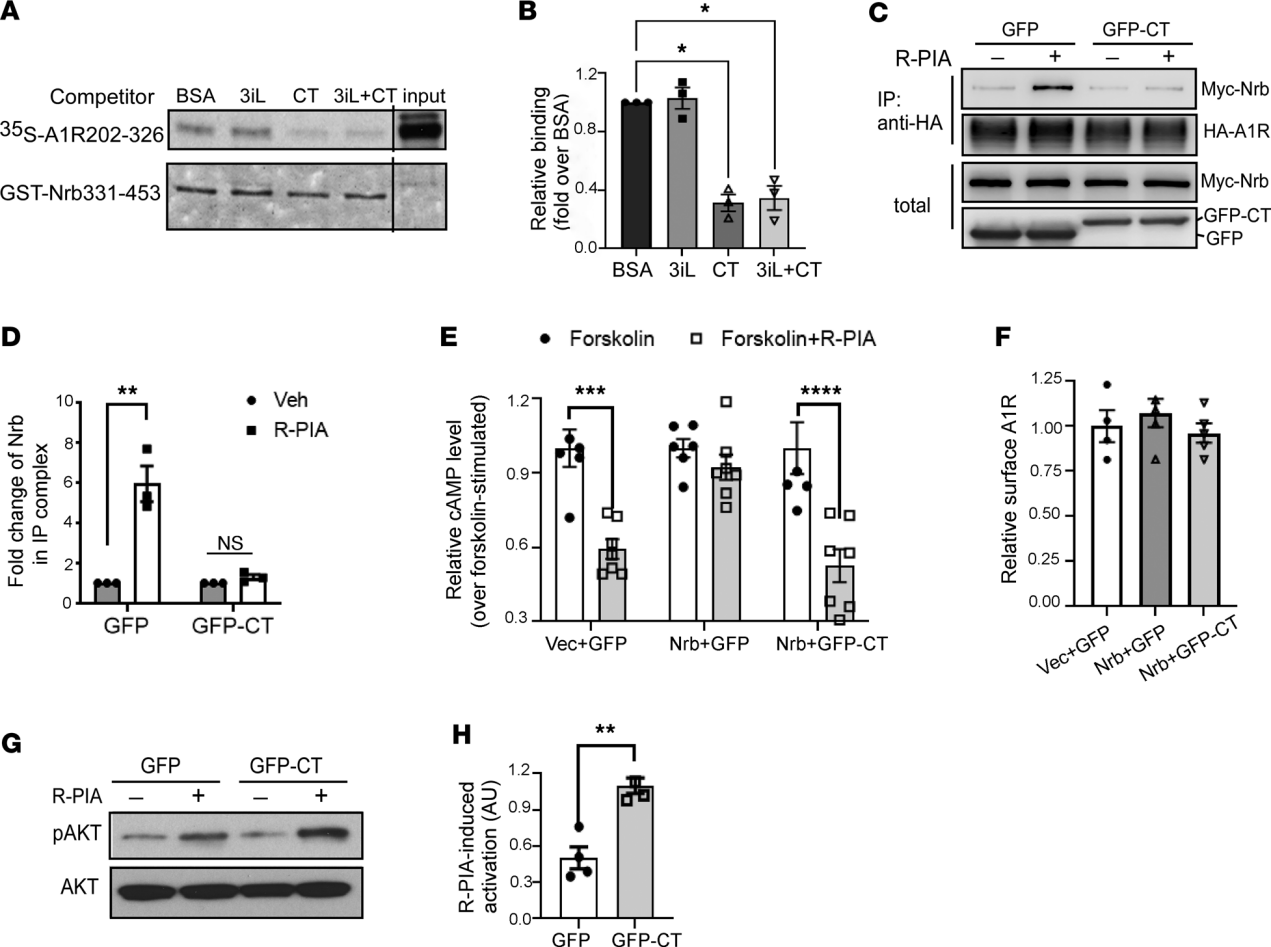
The A1R-CT peptide blocks the A1R-neurabin interaction and restores A1R-mediated signaling in the presence of neurabin. (**A**) Competition pull-down assays testing the abilities of A1R 3i loop (3iL) and CT peptides in blocking the interaction between GST-Nrb331-453 and [^35^S]-labeled A1R202-326. Representative autoradiograph and Coomassie staining are shown. (**B**) Quantitation of the amount of [^35^S]A1R202-326 pulled down by GST-Nrb331-453. Free probe represents 1/10 of the input in each reaction. *P < 0.05 versus BSA control by 1-way ANOVA Dunnett’s multiple comparisons test. *n* = 3/group. (**C**) Expression of GFP-CT abolished agonist-induced interaction between neurabin and A1R in intact cells. Representative Western blots are shown. (**D**) Quantitation of the change of neurabin in the IP complex with HA-A1R. **P < 0.01; ns, no significance, R-PIA versus vehicle by 2-way ANOVA Sidak’s multiple comparisons test. *n* = 3/group. (**E**) Expression of GFP-CT restored A1R-mediated inhibition of cAMP production in the presence of neurabin expression. Cells stably expressing HA-A1R were cotransfected with vectors encoding indicated proteins. Vec, empty vector. Cells were treated with 10 μM forskolin alone or forskolin plus 1 μM R-PIA. Data are expressed as fold change in cAMP production over forskolin alone control. ***P < 0.001; ****P < 0.0001; forskolin+R-PIA versus forskolin by 2-way ANOVA Sidak’s multiple comparisons test. *n* = 6 for forskolin and *n* = 7 for forskolin+R-PIA in each set. (**F**) Expression of GFP-CT does not alter the cell surface level of A1R. *n* = 4/group. (**G**) Expression of GFP-CT enhances A1R-mediated Akt activation. Cells stably expressing HA-A1R with GFP or GFP-CT were stimulated with vehicle or 1 μM R-PIA. Representative blots are shown. (**H**) Quantitation of pAkt activation. The quantitative value of pAkt was normalized by that of Akt blotted on the same gel or on a separate gel that was run in parallel contemporaneously. **P < 0.01 by 2-tailed Student’s *t* test. *n* = 4/group. Error bars represent mean ± SEM.

Our in vitro and in vivo data described above collectively suggest that targeting the A1R-neurabin interaction represents an attractive strategy to specifically enhance A1R-mediated anti-seizure and neuroprotective effects. Therefore, we searched for blockers that could disrupt the direct interaction between A1R and neurabin. The C-tail and 3i loop of A1R are both involved in binding with neurabin aa331-453 (Nrb331-453) (21, 22). We synthesized peptides that carry the sequences of the A1R-CT (aa293-326) and 3i loop (aa202-235), respectively, and tested their ability to disrupt the A1R-neurabin interaction. The addition of the A1R-CT peptide markedly reduced the direct interaction between the A1R (aa202-326) with Nrb331-453 in in vitro pull-down assays (Figure 4, A and B). Conversely, the presence of the 3i loop peptide had no effect on the A1R-neurabin interaction (Figure 4, A and B). These data suggest that the A1R-CT peptide acts as an effective blocker to disrupt the direct interaction between A1R and neurabin. This notion is further supported by data collected using intact cells, where expression of GFP-fused A1R-CT (GFP-CT) abolished the interaction between the full-length A1R and neurabin (Figure 4, C and D).

We next examined the effect of the A1R-CT peptide on A1R signaling. In CHO cells, in which endogenous neurabin expression could not be detected, expression of exogenous neurabin diminished A1R-induced inhibition of cAMP (Figure 4E). When GFP-CT was coexpressed with neurabin, A1R-mediated cAMP inhibition was fully restored (Figure 4E). Expression of GFP-CT did not alter surface expression of A1R (Figure 4F). We also tested the effect of GFP-A1R-CT on A1R-mediated Akt activation. In cells overexpressing GFP-A1R-CT, the level of Akt phosphorylation in response to A1R agonist R-PIA was significantly enhanced (Figure 4, G and H). Taken together, these data suggest that the neurabin-mediated attenuation of A1R signaling can be abolished by the expression of the A1R-CT peptide. Therefore, we identified a peptide blocker (i.e., the A1R-CT peptide) that effectively disrupts the A1R-neurabin interaction and enhances A1R signaling.

### The A1R-CT peptide displays prominent protective effects against chemically induced seizures.

**Figure 5:**
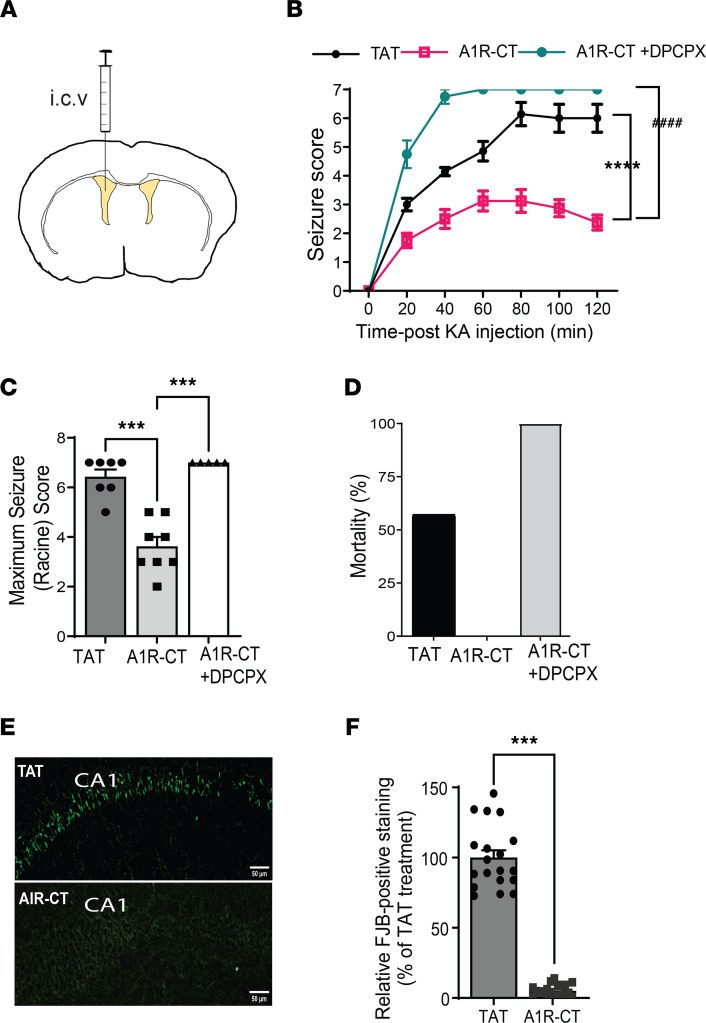
The A1R-CT peptide displays robust anticonvulsant and neuroprotective effects in an A1R dependent manner. (**A**) A schematic diagram indicates the TAT-fused A1R-CT peptide or TAT control peptide infused into the left ventricle 30 minutes prior to kainate injection. (**B**) Seizure severity in response to kainate is measured in mice with indicated treatment. ****P < 0.0001, A1R-CT versus TAT; ^####^*P* < 0.0001, A1R-CT versus A1R-CT+DPCPX, by 2-way ANOVA. *n* = 7 for TAT and A1R-CT+DPCPX; *n* = 8 for A1R-CT. (**C**) Quantitation of maximum seizure scores in mice with indicated treatment. ***P < 0.01 by 1-way ANOVA Tukey’s multiple comparisons test. (**D**) Kainate-induced lethality is recorded in mice examined in B. Data are expressed as percentage of death. (**E**) Representative images of cell death in the hippocampal CA1 region as revealed by Fluoro-Jade B (FJB) staining. Scale bar: 100 μm. (**F**) Quantitation of the CA1 neurons with positive FJB staining. Data are expressed as the percentage of the level in TAT-treated mice (which is set as 100%). ***P < 0.001 by paired 2-tailed Student’s *t* test. *n* = 19 slices from 3 mice for TAT and *n* = 19 slices from 3 mice for A1R-CT. Data are expressed as mean ± SEM.

Our goal is to improve A1R-elicited anti-seizure function in the brain. We next sought to determine whether the A1R-CT peptide could enhance A1R-mediated anti-seizure effects in a kainate-induced model. The A1R-CT peptide was synthesized with addition of the Trans-Activator of Transcription (TAT) peptide sequence to facilitate diffusion into cells. The TAT-A1R-CT or TAT control peptide (500 pmole) was infused into the brain through an i.c.v. cannula 30 minutes prior to kainate injection (Figure 5A). In mice receiving the TAT-A1R-CT peptide, the severity and duration of kainate-induced seizures were significantly reduced compared with those in mice receiving the TAT peptide (Figure 5, B and C). While 57% of mice receiving the TAT peptide died within 2 hours after kainate injection, all mice receiving the TAT-A1R-CT peptide survived (Figure 5D). When mice were cotreated with DPCPX, the anti-seizure effect of A1R-CT was no longer observed (Figure 5, B–D), suggesting the requirement of A1R in mediating the protection by this peptide.

We further examined neuronal death in the hippocampus of mice that survived kainate insult. Severe cell death was observed in the hippocampus of mice receiving the TAT peptide 7 days after kainate-induced seizures (Figure 5, E and F). However, in hippocampal slices prepared from TAT-A1R-CT peptide–treated mice exposed to the same level of kainate injections, little cell death was detected (Figure 5, E and F). These morphological findings of neuronal cell survival, paralleled with the seizure and survival findings shown above (Figure 5, B–D), demonstrate a prominent neuroprotective effect of the A1R-CT peptide against excitotoxic cell death.

**Figure 6:**
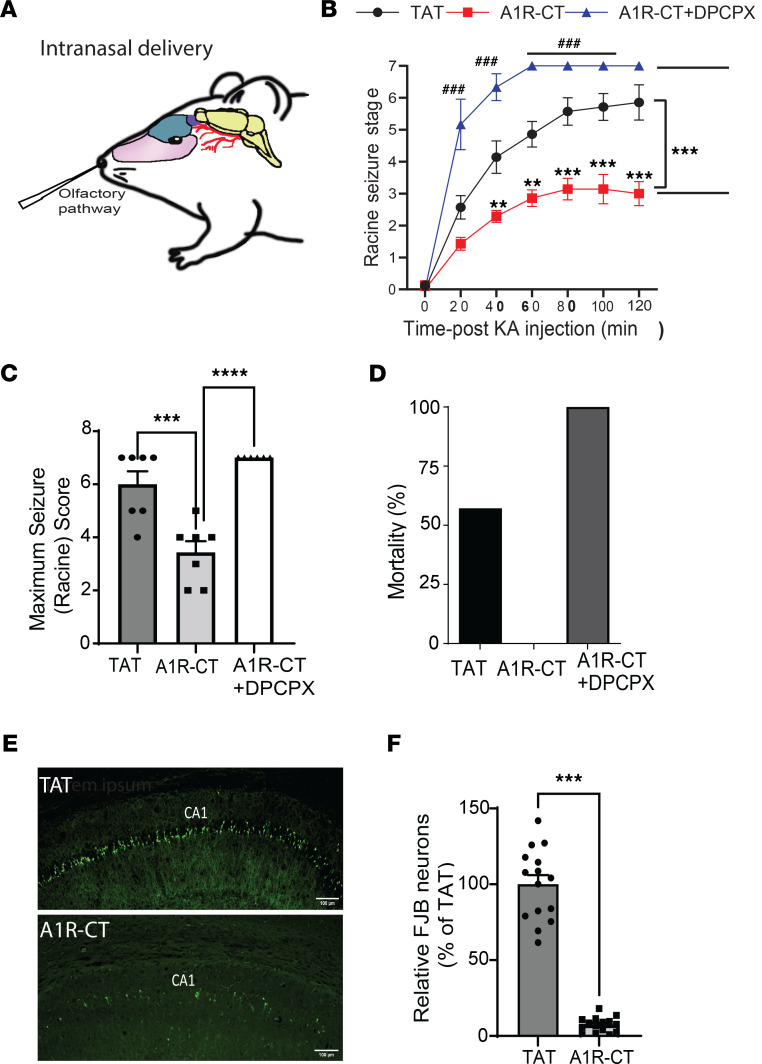
Intranasal delivery of the A1R-CT peptide shows effective anti-seizure and neuroprotective effects. (**A**) A schematic diagram indicates the A1R-CT peptide (fused with TAT) or TAT control peptide administered into the nasal cavity. (**B**) Seizure severity in response to kainate is measured in mice with the indicated treatment. **P < 0.01, ***P < 0.001, A1R-CT versus TAT; ^###^*P* < 0.001, A1R-CT versus A1R-CT+DPCPX by 2-way ANOVA Tukey’s multiple comparisons test. *n* = 7 for TAT and A1R-CT; *n* = 6 for A1R-CT+DPCPX. (**C**) Quantitation of maximum seizure scores in mice with the indicated treatment. ***P < 0.001, A1R-CT versus TAT; ****P < 0.0001, A1R-CT versus A1R-CT+DPCPX by 1-way ANOVA Tukey’s multiple comparisons test. (**D**) Kainate-induced lethality is recorded in mice examined in B. Data are expressed as percentage of death. (**E**) Representative images of cell death in the hippocampal CA1 region as revealed by Fluoro-Jade B (FJB) staining. Scale bar: 100 μm. (**F**) Quantitation of the CA1 neurons with positive FJB staining. Data are expressed as the percentage of the level in TAT-treated mice (which is set as 100%). ***P < 0.001 by paired 2-tailed Student’s *t* test. *n* = 15 slices from 3 mice for TAT and *n* = 15 slices from 3 mice for A1R-CT. Data are mean ± SEM.

Peptides delivered i.n. rapidly reach the brain in a noninvasive way. We tested whether i.n. administration of the TAT-A1R-CT peptide could also elicit anti-seizure effects. The TAT-A1R-CT or TAT peptide (42 nmole) was delivered into the i.n. cavity 45 minutes prior to kainate injection (Figure 6A). In mice receiving the TAT-A1R-CT peptide, the severity and duration of kainate-induced seizures were significantly reduced when compared with those in mice receiving the TAT peptide (Figure 6, B and C). All mice receiving the TAT-A1R-CT peptide survived, in contrast with the 60% death rate caused by kainate in mice receiving the TAT peptide (Figure 6D). The observed anti-seizure effect of the TAT-A1R-CT peptide was abolished by cotreating mice with DPCPX (Figure 6, B–D), suggesting that A1R is required to mediate the protection by this peptide. Furthermore, while kainate insult resulted in severe cell death in the hippocampus of mice receiving the TAT peptide, only limited neuronal death was detected in mice receiving the TAT-A1R-CT peptide (Figure 6, E and F). These data clearly demonstrate the effectiveness of i.n. delivery of the A1R-CT peptide in protection against chemoconvulsant seizures and excitotoxic cell death.

### The A1R-CT peptide reduces spontaneous epileptic activity in an AD mouse model.

**Figure 7:**
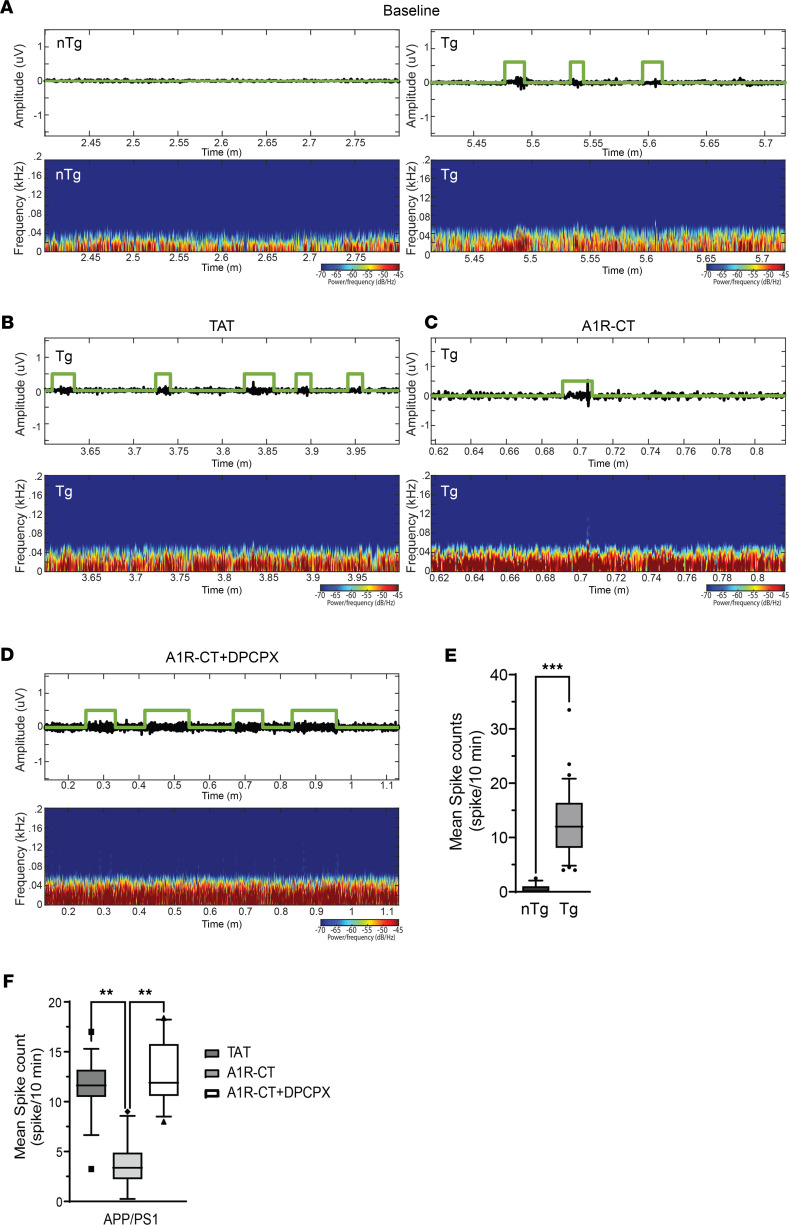
Intranasal delivery of the A1R-CT peptide effectively reduces epileptic activities in the brain of APP/PS1 mice. Eleven-month-old APP/PS1 and nTg littermates were subjected to EEG recording. (**A**) Representative EEG trace and spectrogram in nTg and APP/PS1 mice at baseline. (**B–D**) Representative EEG trace and spectrogram in APP/PS1 mice after TAT (**B**) or TAT-fused A1R-CT (**C**) peptide treatment or A1R-CT+DPCPX treatment (**D**). (**E**) Quantitation of spike frequency with repeated measurement in nTg and APP/PS1 mice at baseline. Data were obtained from analyzing EEG recordings of 3 nTg and 3 APP/PS1 mice. (**F**) Quantitation of spike frequency with repeated measurement in APP/PS1 mice with indicated treatments. Data were obtained from analyzing EEG recordings of 4 TAT-treated, 4 A1R-CT–treated and 5 A1R-CT+DPCPX–treated APP/PS1 mice. Box-and-whisker plots represent median and 5–95 percentile range of all measurements for each group. **P < 0.01, ***P < 0.001 by a repeated measure 1-way ANOVA used to calculate the statistical difference of the spike count per 10 minutes between nTg and APP/PS1 mice and among different treatments in APP/PS1 mice.

The above data demonstrate that the A1R-CT peptide provides protection against chemoconvulsant seizures. Next, we sought to determine its effectiveness in reducing spontaneous epileptic activities in disease models. We decided to examine an AD mouse model, as most current antiepileptic drugs have adverse effects on cognition or mood, making treatment of seizures in AD patients particularly challenging. We chose to test amyloid precursor protein/presenilin 1 (APP/PS1) transgenic mice using EEG recording. These mice start to develop spontaneous seizures at the onset of amyloid pathogenesis (27). We monitored EEG activities in freely moving 10- to 11-month-old APP/PS1 mice and their nontransgenic (nTg) littermates. APP/PS1 mice develop spontaneous epileptic activities as manifested by the appearance of epileptic spikes, and such electrographic paroxysms were not observed in their nTg littermates (Figure 7, A and E).

We then treated APP/PS1 mice with the TAT or TAT-A1R-CT peptide through i.n. delivery, with or without i.p. injection of DPCPX, and continuously monitored their brain activities for 18 hours. The EEG recording of APP/PS1 mice treated with the TAT peptide displayed similar electrographic paroxysms as observed in the baseline recording of these mice (Figure 7B). However, treatment with the TAT-A1R-CT peptide led to a significant reduction in the number of epileptic spike counts compared with TAT peptide treatment in APP/PS1 mice (Figure 7, C and F). Delivery of the TAT-A1R-CT peptide i.n. failed to reduce epileptic activities in APP/PS1 mice that also received DPCPX (Figure 7, D and F), suggesting the requirement of A1R in mediating the antiepileptic effect of the TAT-A1R-CT peptide. These data suggest that the A1R-CT peptide can effectively inhibit spontaneous seizure activities associated with AD.

## Discussion

In the present study, we identified a potentially novel blocking peptide, the A1R-CT peptide, that exhibits strong protective effects against both chemoconvulsant seizures and spontaneous epileptic activities in an AD mouse model. This blocking peptide disrupts the direct interaction between A1R and neurabin, a neural tissue-specific protein that negatively regulates A1R signaling and function (21, 22). As a result, the A1R-CT peptide enhances A1R-mediated signaling responses and boosts endogenous adenosine-induced anti-seizure effects through A1R. Of particular significance, in all experiments performed in our study, no exogenous A1R ligands were administered. The anticonvulsant and neuroprotective effects of this peptide blocker are achieved through the A1R in response to the endogenous adenosine released on-site, thus providing precise protection at the zone of hyperexcitability. Furthermore, neurabin is a neural-specific scaffolding protein. Therefore, this agent elicits its beneficial effects against seizures without inducing confounding outcomes due to ectopic activation of receptors in peripheral tissues and organs.

Pathological conditions such as hypoxia, ischemia, and excitotoxin exposure result in hyperexcitability and cell death of neurons in the brain. The severity of neural damage is a key factor determining mortality and morbidity under these conditions. One key endogenous defense mechanism against these insults is the release of extracellular adenosine from glia and neurons, which acts on A1R to reduce neural damage and to confine the development and progression of accompanying seizures that result from hyperexcitability (9–12, 28, 29). The current study is based on our previous discovery of a key role of the A1R/neurabin/RGS4 complex in controlling A1R signaling strength (21, 22); neurabin forms a homodimer, with 1 monomer binding A1R and the other binding RGS4, to turn off A1R-induced G protein activation (22). Here we show that A1R-mediated synaptic inhibition (Figure 2) is significantly enhanced in the absence of neurabin or RGS4 expression. As a result, neurabin- or RGS4-deficient mice show resistance to chemoconvulsant seizures in an A1R-dependent manner (Figure 1). A particularly striking phenomenon is that a 50% drop of neurabin expression leads to a significant reduction in seizure severity (Figure 1), suggesting that endogenous adenosine-elicited anticonvulsant effects through A1R would be sensitive to modulation of the A1R-neurabin interaction. In addition, we find that A1R-mediated neuroprotective Akt signaling is both enhanced and prolonged in the absence of neurabin (Figure 3). These findings motivated us to search for blockers that could disrupt the A1R-neurabin interaction, which would serve as an effective means to enhance A1R-mediated anti-seizure and neuroprotective effects.

Our previous structure-function analysis has revealed that the interaction interface between A1R and neurabin involves both the 3i loop and CT of the receptor (21, 22). In this study, we demonstrate that the peptide consisting of the A1R-CT sequence, but not the 3i loop sequence, effectively blocks the direct interaction between A1R and neurabin and reverses neurabin-mediated inhibition of A1R signaling (Figure 4). Significantly, when directly delivered into the brain, this A1R-CT peptide shows strong anti-seizure and neuroprotective effects in an A1R-dependent fashion (Figure 5), providing direct evidence for the effectiveness of blocking neurabin-A1R interaction as a means for seizure control. It is worth noting that the region of neurabin that interacts with A1R does not overlap with other functional domains of neurabin (21); thus, the A1R-CT peptide is unlikely to alter neurabin functions in other processes.

To demonstrate the strong potential of this peptide as a therapeutic agent for seizure control, we show that noninvasive i.n. delivery of this peptide displays robust anti-seizure and neuroprotective effects against kainate (Figure 6) and reduces epileptic spikes in an AD mouse model. Currently available anticonvulsant drugs act through activation of GABAergic transmission or inhibition of Na^+^ or Ca^2+^ channels. If successfully developed for human use, the A1R-CT peptide would represent the first to offer a different and more targeted therapy facilitating seizure termination. The i.n. delivery of this peptide may also provide a potentially new option for seizure rescue treatments. Currently, there are only 3 seizure rescue medications: diazepam rectal gel (Diastat), diazepam nasal spray (Valtoco), and midazolam nasal spray (Nayzilam), the latter 2 of which were just approved by the FDA in 2020.

In addition to being caused by acute insults to the brain, epileptic seizures are a common comorbidity of chronic neurodegenerative diseases, including AD, the most common type of dementia. Dementia of AD type is, in fact, more commonly associated with seizure than non-AD dementia (30, 31). Epileptic activities can occur at the early stages of AD, concurrently with or even preceding the onset of cognitive decline (32, 33). The incidence of seizures increases with the disease progression (4, 34, 35). In addition, seizure occurrence exacerbates cognitive deficits in AD (36, 37), while anti-seizure medication improves cognition (38). Treatment of seizures in patients with AD remains a challenging task since most current ASDs have adverse effects on cognition or mood (3). Here we examined the effectiveness of the A1R-CT peptide in suppressing seizures in an AD animal model, APP/PS1 mice. Similar to human patients, AD animal models display network hyperexcitability and seizures (39, 40). Our data clearly demonstrate that i.n. delivery of the A1R-CT peptide effectively reduces the spontaneous spike frequency in the APP/PS1 model (Figure 7). This study thus provides strong preclinical evidence supporting that noninvasive delivery of the A1R-CT peptide is effective in treating AD-related seizures. Whether the A1R-CT peptide can also reduce the progression of AD-related pathology and improve cognition is currently under investigation.

In summary, we developed a potentially novel peptide blocker of the A1R-neurabin interaction, the A1R-CT peptide, which displays strong protective effects against both chemoconvulsant and AD-related spontaneous seizures. The anti-convulsant and neuroprotective effects of this peptide are in response to the endogenous adenosine released on-site and on-demand, thus avoiding ectopic activation of receptors in other tissues and organs. Thus, the A1R-CT peptide represents a promising therapeutic intervention for seizure control under various pathological conditions. The demonstrated effectiveness of i.n. delivery of this agent makes it particularly attractive and clinically relevant as a potential novel seizure-rescue treatment. Furthermore, to the best of our knowledge, this peptide represents the first agent that specifically enhances A1R functions in the CNS and would be useful for the treatment of other neurological disorders in which A1R function is warranted.

## Methods

### Peptides, Abs, and chemicals.

The TAT alone and TAT-fused A1R-CT peptides were synthesized by GenScript. The A1R-3iloop and A1R-CT peptide without TAT fusion were synthesized by American Peptide. Rabbit monoclonal phospho-Akt (Thr308) (model 244F9, catalog 4056S), mouse monoclonal Akt (catalog 2920S) and mouse monoclonal Myc-Tag (model 9B11, catalog 2276S) antibodies were purchased from Cell Signaling Technology. Mouse HA.11 was purchased from BioLegend (catalog MMS-101), rat anti-HA was from Roche (catalog 1815016), and rabbit Neurabin 1 (PPP1R9A) antibody was from Epitomics (catalog 3717-1). [^35^S]Methionine was purchased from PerkinElmer. R-PIA ([−]-N6-[2-Phenylisopropyl] adenosine), yohimbine, glutamate, forskolin, and LY294002 were from Sigma. Kainite and Fluoro-Jade B were purchased from Milestone Pharmatech and Fisher Scientific. All other chemicals were from Sigma-Aldrich or Fisher Scientific.

### Animals.

All mice were housed in the AAALAC-accredited Animal Resources Program facility at the University of Alabama at Birmingham in accordance with procedures of the Animal Welfare Act and the 1989 amendments to the Act, and all studies followed protocols approved by the UAB Institutional Animal Care and Use Committee. Generation of the RGS4-deficient (*Rgs4^–/–^*) mouse line has been described previously (24). *Rgs4^–/–^* mice were provided by Venetia Zachariou (Mount Sinai School of Medicine, New York, NY) and compared with their corresponding WT mice at the same age. Neurabin-deficient (*Ppp1r9a^–/–^*) mice were described previously (21, 41) and provided by Paul Greengard (Rockefeller University, New York, NY). Neurabin WT, heterozygous, and homozygous littermates were used in this study. APP/PS1 transgenic mice on the C57BL/6 background were described previously (42, 43) and provided by Ling Li (University of Minnesota, Minneapolis, MN). Gender-matched littermate APP/PS1 and nTg controls (10- to 11-months-old) were used. Both male and female mice were used, and data were combined, as no significant sex difference was observed. All mice were backcrossed to and maintained on the C57BL/6 genetic background for over 10 generations.

### Induction and evaluation of chemoconvulsant seizures.

Induction and evaluation of seizures were performed as described previously (21). Male mice (10- to 12-weeks-old) were injected i.p. with 20 or 25 mg/kg of kainate (Sigma) dissolved in saline, together with or without DPCPX (Sigma, 0.5 mg/kg in saline) or yohimbine (Sigma, 0.5 mg/kg in saline). Seizure severity was scored by trained observers blind to genotype and/or treatment. The severity of seizure behaviors was scored on a scale of 0–7, with 0 representing normal behavior and 7 representing death (44, 45). To induce seizures, mice were injected i.p. with 40 mg/kg of PTZ dissolved in saline, with or without DPCPX. Seizure severity was scored every 4 minutes for 30 minutes.

### Acute hippocampal slice preparation and electrophysiology.

Mice (8- to 12-weeks-old) with indicated genotypes were anesthetized with isoflurane. Brains were removed and dissected into coronal slices (350 μm thick) from dorsal hippocampus on a vibratome (Leica Biosystems, VT1000P) using ice-cold high sucrose cutting solution (in mM as follows: 85.0 NaCl; 2.5 KCl; 4.0 MgSO_4_; 0.5 CaCl_2_;1.25 NaH_2_PO4; 25.0 glucose; and 75.0 sucrose), oxygenated with 95% O_2_ and 5% CO_2_. After sectioning, slices were transferred to artificial cerebral spinal fluid (ACSF) containing the following (in mM): 119.0 NaCl; 2.5 KCl; 1.3 MgSO_4_; 2.5 CaCl_2_; 1.0 NaH_2_PO_4_; 26.0 NaHCO_3_; and 11.0 glucose. Slices were held from 1–5 hours in a submersion chamber continuously bubbled with 95% O_2_ and 5% CO_2_ at room temperature.

Hippocampal slices were placed in the recording chamber, with continuous perfusion of gassed ACSF at a constant rate (2 mL/min) at room temperature. Extracellular fEPSPs were recorded in the stratum radiatum using a glass pipette filled with ACSF. A bipolar stimulating electrode was placed in CA1 stratum radiatum to stimulate Schaffer collateral axons (0.1 Hz, 100 μs duration). Baseline fEPSPs were obtained at 80–180 μA to elicit baseline fEPSPs of 0.4–0.6 mV in amplitude (40–50% maximal field amplitude) for 20 minutes. Experiments were excluded if there was more than 8% variance in baseline. Extracellular CA1 pyramidal population spikes (PSs) were evoked using a stimulating electrode positioned in CA1 stratum radiatum to stimulate Schaffer collateral axons at 0.1 Hz with 100 μs duration and recorded using a glass micropipette placed in stratum pyramidale. Data were collected using an Axopatch 1D amplifier (Molecular Devices) in current-clamp mode, and signals were filtered at 2 kHz and acquired using pCLAMP 10.2 acquisition software (Molecular Devices).

### Measurement of cAMP levels.

cAMP assays were performed using AlphaScreen Assay Kit from PerkinElmer following the manufacturer’s instruction. In brief, cultured CHO cells were cotransfected with cDNA encoding HA-A1R, myc-neurabin, and GFP or GFP-A1R293-326. Cells were washed once and collected in PBS 48 hours following transfection. The pellet was then resuspended with the stimulation buffer (1× HBSS; 0.1% BSA; 0.5 mM IBMX; 5 mM HEPES; and pH7.4) and mixed with anti-cAMP acceptor beads. The mix was divided into 3 groups with the following treatment: (a) vehicle; (b) 10 μM forskolin; and (c) 10 μM forskolin and 5 μM R-PIA. A total of 20 minutes after stimulation at 37^o^C, biotinylated cAMP/streptavidin donor beads in lysis buffer (0.1% BSA; 0.3% Tween20; 5mM HEPES; and pH7.4) was added to cells/acceptor beads mix. After 30 minutes of incubation at room temperature, luminescence was analyzed on a Biotek Synergy2 plate reader using standard α-screen settings.

### Intact cell surface ELISA.

Cell surface HA-A1R expression in CHO-K1 cells transfected with GFP or GFP-A1R-CT was examined by the cell-surface ELISA method as described previously (21, 46). In brief, cells were fixed and then subjected to blocking, primary Ab (HA11, 1:3000), and secondary Ab (HRP-conjugated anti-mouse, 1:2000). Following incubation with o-phenylenediamine substrate (Pierce), absorbance at 490 nm was measured to determine surface HA-A1R density.

### In vitro GST pull-down.

Preparation of GST fusion proteins, synthesis of [^35^S]-labeled in vitro translated probes, and pull-down assays were performed as described previously (21, 22). The A1R 3i loop (aa202–235) and CT (aa293–326) peptides used for blocking A1R/neurabin interaction were synthesized at Peptide 2.0 and dissolved in water with the stock concentration at 5 μg/μL. The peptide concentration in the final pull-down assay was 0.1 μg/μL.

### Cells and transfection.

All cell lines were obtained from American Type Culture Collection (ATCC). CosM6 cells were cultured in DMEM (Life Technologies) supplemented with 10% FBS (Atlanta Biologicals), 100 U/mL penicillin, and 10 μg/mL streptomycin. CHO-K1 cells were cultured in DMEM/F-12 (Life Technologies) with 10% FBS, penicillin and streptomycin, and 2 mM glutamine. Neuro2A cells were cultured in 1:1 DMEM/Opti-MEM mix (Thermo Fisher Scientific) supplemented with 5% FBS, 100 U/mL penicillin, and 100 μg/mL streptomycin. Cells were transfected with Lipofectamine 2000 (Life Technologies) following manufacturer’s instruction.

### Primary hippocampal culture and glutamate-induced cell death.

Primary hippocampal culture was performed as we described previously (21). To examine the neuroprotective effect of the A1R agonist R-PIA, we induced neuronal death with glutamate under varying conditions and then determined cell viability using LIVE/DEAD Viability/Cytotoxicity kit (Life Technologies) following the manufacturer’s instruction. In brief, hippocampal neurons were cultured in 24-well plates for 13–14 days in vitro and treated with (a) vehicle; (b) 100 μM glutamate alone; (c) 100 μM glutamate plus 10 μM R-PIA; and (d) 100 μM glutamate, 10 μM R-PIA, and 30 μM LY294002 for 30 minutes at 37°C. Neurons were then washed twice and incubated with regular growth medium at 37°C for an additional 24 hours. Neurons were stained with 4 μM calcein-AM and 4 μM EthD-1 in DPBS and mounted for imaging under a fluorescence microscope.

### Cannulation and intracerebroventricular infusion of peptide.

Stainless-steel single guided cannulas (26 gauge, RWD Life Science) were implanted into the lateral ventricles with ± 1.0, –0.3, and –2.3 (x, y, z) under isoflurane anesthesia using standard stereotaxic procedures. Coordinates were chosen based on the mouse brain atlas. The cannula was anchored to the skull using screws and acrylic cement. Mice were allowed to recover for 7–10 days after surgery. The injection cannula was connected via PE Tubing (1.50 × 0.50 mm; RWD Life Science) to a 10 μL Hamilton microsyringe, driven by a microinjection pump (Dual Syringe, Model ‘11’, Harvard apparatus, MA-70-2209). Infusions were administered in a volume of 5 μL over 10 minutes, and an additional 1 minute was allowed for diffusion before the infusion cannulas were removed. The A1R-CT or TAT control peptide (500 pmole in 5 μL) were administered 30 minutes prior to i.p. injection of kainate or kainate plus DPCPX.

### Peptide i.n. administration.

Mice were habituated for a few days (gripping, scruffing, and positioning) before peptide administration as described previously (47). For peptide delivery, mice were held and positioned with the neck and chin flat, and the pipette tip holding the peptide solution was placed at a 45° angle, following a procedure described previously (47). A total of 6 μL of peptide solution (7.0 mM) was slowly administered to 1 nostril with 2–3 intervals, and the mouse was held in position for an additional 5 seconds after the last drop. The administration step was repeated for the other nostril. The total volume injected (12 μL) is less than that of the mouse nasal cavity which is approximately 32.5 mm^3^. Peptides were administered 45 minutes prior to i.p. injection of kainate.

### Assessment of cell death in hippocampal slices.

A total of 7 days after initial kainate exposure, mice were deeply anesthetized with isoflurane and perfused with 4% paraformaldehyde in PBS, pH 7.4. Hippocampal slices (30 μm) were stained with Fluoro-Jade B staining following the manufacturer’s instructions.

### EEG recordings and analysis.

Implantation of electrodes for EEG recordings was performed under isoflurane anesthesia (4.5% for induction, ~ 2% for maintenance). In brief, 1 mm stainless steel electrodes (P1 Technologies) were implanted into the primary somatosensory cortex at 1.8, 0.02, and 1 (AP, ML, and DV, respectively), and reference electrodes anterior to the bregma were implanted subdurally through small holes drilled in the skull. These electrodes were held in place with Dental cement kit (Stoelting) and C&B Metabond Quick Adhesive Cement System (Parkell). The ground electrode was sutured in the cervical paraspinous area. All electrodes were inserted into a 6-channel pedestal and connected to the commutator for recording (48). Mice were allowed to recover for 5–7 days after the operation. EEG activity was recorded for 20 hours using Biopac Systems amplifiers (Biopac Systems, EEG100C) and AcqKnowledge 4.2 EEG Acquisition and analyzed with the Reader Software (Biopac Systems).

A total of 150 hours of data was analyzed, 54 hours for nTg mice and 96 hours for APP/PS1 transgenic mice. A total of 6 hours of recording were analyzed for each animal. All signals were filtered with a FIR notch filter at 60Hz and its corresponding harmonics and with a bandpass filter from 0.1 to 150Hz. Spikes were defined as a sharp amplitude deflection lasting 20–70 ms. EEG analysis was performed using MATLAB (Mathworks, 2020a). Signals were clipped into 10-minute segments and determination of epileptiform activity and quantification of interictal spike activity were performed using a semiautomated analysis with p-operator algorithms described in (49, 50). P-operator algorithm was used with a sliding window of 1 second. When p-operator algorithm detected a spike, all signals were visually inspected to include in the analysis only those that were true positives in order to avoid nonepileptic artifacts caused by electrical noise (e.g., grooming) during spike selection. Spectral content of EEG signals was calculated using short-time Fourier transform (FFT).

### Statistics.

Seizure scores were analyzed by 2-way ANOVA with multiple comparisons test. Statistical analyses were performed using Prism GraphPad software. For EEG data, a repeated measure ANOVA was used to calculate the statistical difference of the spike count per 10 minutes among different treatments between nTg and APP/PS1 mice. Statistical tests were performed on IBM SPSS. Other data were analyzed with Student’s *t* tests, 1-way or 2-way ANOVA with multiple comparisons test using GraphPad software. *P* < 0.05 was considered statistically significant.

### Study approval.

Experimental procedures were carried out with the approval of, and in accordance with, the Animal Resources Program and Institutional Animal Care and Use Committee at the University of Alabama at Birmingham.

## Author contributions

QW and KJ conceived the study. SS, YC, and LC performed experiments and analyzed data. WJL and SP contributed to data acquisition or analysis. DP analyzed EEG recording data. LM, KJ, and QW were involved in experimental design and data analysis. SS, YC, LC, DP, SP, LM, KJ, and QW prepared the manuscript. SS and YC contributed equally to the original study. SS also contributed to the revision and is, therefore, listed first.

## Supplementary Material

Supplemental data

## Version 1 06/08/2022

Electronic publication
