# Supplementary material for: A peptide blocking the ADORA1-neurabin interaction is anticonvulsant and inhibits epilepsy in an Alzheimer’s model
Source: JCI Insight. 2022 Jun 8;7(11):e155002. doi: 10.1172/jci.insight.155002 (PMC9220929; doi:10.1172/jci.insight.155002)

Full unedited gels

Unedited gels for Figure 1F, top to bottom

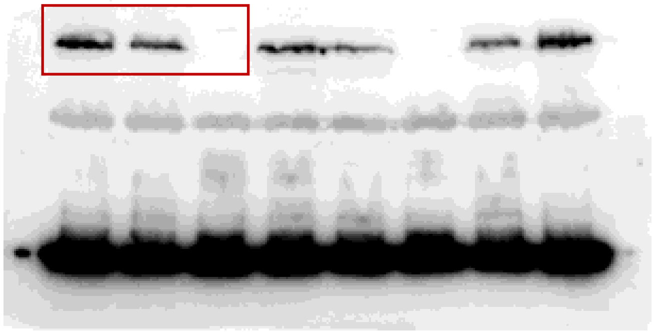

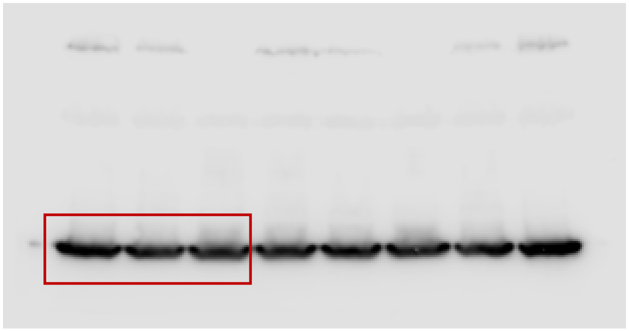

Unedited gels for Figure 3C, top to bottom

Unedited gels for Figure 3E, top to bottom

Unedited gels for Figure 4A, top to bottom

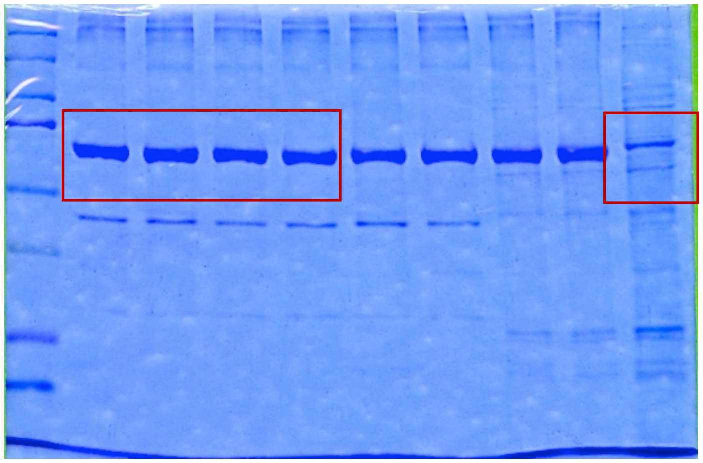

Unedited gels for Figure 4C, top to bottom

Row 1 Row 3

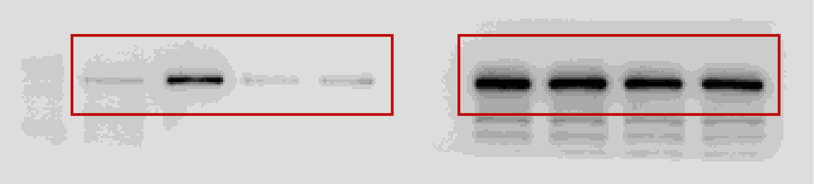

Row 2

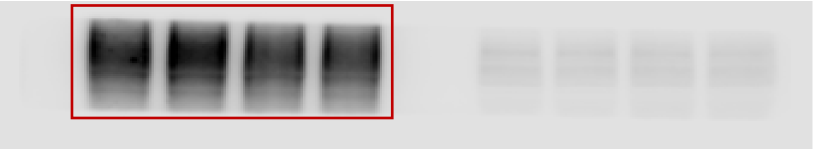

Row 4

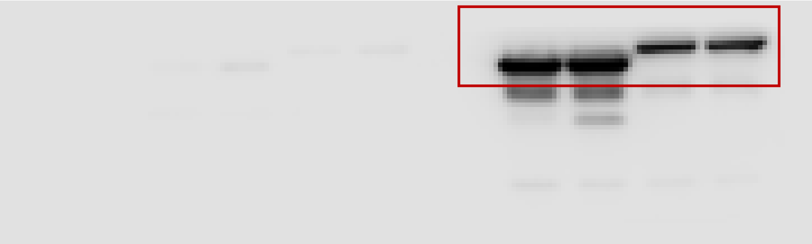

Unedited gels for Figure 4G, top to bottom

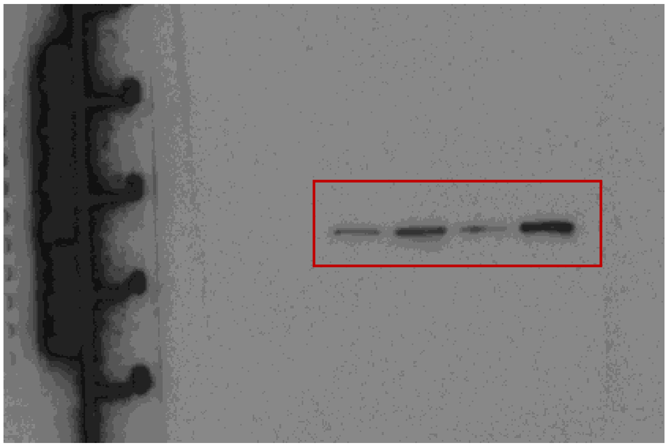

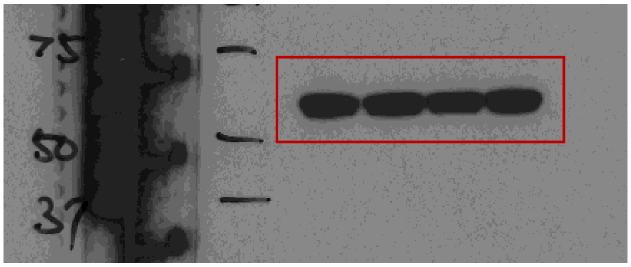

Supplement: Supplemental data [file jciinsight-7-155002-s171.pdf]
